# Spatiotemporal Coupling of DNA Supercoiling and Genomic Sequence Organization—A Timing Chain for the Bacterial Growth Cycle?

**DOI:** 10.3390/biom12060831

**Published:** 2022-06-15

**Authors:** Georgi Muskhelishvili, Patrick Sobetzko, Andrew Travers

**Affiliations:** 1 School of Natural Sciences, Biology Program, Agricultural University of Georgia, 0159 Tbilisi, Georgia; 2 Synmikro, Loewe Center for Synthetic Microbiology, Philipps-Universität Marburg, 35043 Marburg, Germany; sobetzko@staff.uni-marburg.de; 3 MRC Laboratory of Molecular Biology, Cambridge Biomedical Campus, Cambridge CB2 0QH, UK; aat@mrc-lmb.cam.ac.uk

**Keywords:** bacterial growth cycle, growth phase-dependent gene expression, DNA supercoiling, DNA sequence organization, nucleoid-associated proteins

## Abstract

In this article we describe the bacterial growth cycle as a closed, self-reproducing, or autopoietic circuit, reestablishing the physiological state of stationary cells initially inoculated in the growth medium. In batch culture, this process of self-reproduction is associated with the gradual decline in available metabolic energy and corresponding change in the physiological state of the population as a function of “travelled distance” along the autopoietic path. We argue that this directional alteration of cell physiology is both reflected in and supported by sequential gene expression along the chromosomal OriC-Ter axis. We propose that during the *E. coli* growth cycle, the spatiotemporal order of gene expression is established by coupling the temporal gradient of supercoiling energy to the spatial gradient of DNA thermodynamic stability along the chromosomal OriC-Ter axis.

## 1. Introduction

The bacterial, as well as any other cell, is a self-reproducing or autopoietic entity. Accordingly, the growth cycle of the bacterial cell population in batch culture describes a closed, self-reproducing circuit underpinned by an “operationally closed” transcriptional regulation system [1,2,3]. Normally, the growth cycle is initiated by inoculating the stationary cells into a fresh growth medium (aka nutritional shift-up), whereupon the cells start propagation by utilizing the available resources and on exhaustion of the latter, ultimately return to the initial (stationary) physiological state. During the bacterial growth in batch culture, the quality of the growth medium changes, becoming increasingly less nutritious and more poisonous to the cells. This directional alteration in medium quality is paralleled by adaptive changes in cell physiology. Such a sequential traverse of different physiological states by the cell population advancing along the autopoietic path implies the capacity of permanently monitoring the status quo for ongoing adaptation to the changing growth conditions. In other words, the population has to use some kind of continuous or analogue information varying as a function of “travelled distance” along the autopoietic circuit. This view is consistent with the proposed existence in bacteria of some kind of “memory” in the sense of retaining and using information about past events [4]. For example, during chemotaxis, a bacterium is proposed to move directionally by measuring the difference in the fraction of receptors bound in successive intervals of time [5].

## 2. Coupling of Chromosomal Gene Order and Transcription

The continuous information underpinning the operation of the bacterial growth cycle is apparently of a hereditary nature and, therefore, it must be encoded (either itself or its mechanism of production) in the cellular genome. In order to vary concertedly with bacterial growth, this information also has to be dynamic. The genetic information encoded in the form of unique genes is of a discontinuous or digital nature and is static. Therefore, it cannot readily satisfy the demands of continuity and dynamics. However, during the *E. coli* growth cycle the genes, especially those encoding the major regulators of growth and adaptation, are expressed in temporal succession corresponding to their spatial order along the chromosomal replication origin-to-terminus (OriC-Ter) axis. The latter was shown to serve as a coordinate system for genetic regulation [6,7]. Thus, although the underlying gene order is static, the gene expression is subject to control by a dynamic, continuous variable determining the sequential, chromosomal position-dependent gene expression pattern. In this article, we argue that the continuous variable governing the patterns of gene expression at the most fundamental level is the changing genomic distribution of the effective DNA superhelicity [8], defined as the torsional energy available for the untwisting of gene promoters and transcription initiation.

The bacterial chromosome (aka nucleoid) has been proposed to behave as a “smart polymer”, capable of undergoing large conformational transitions (e.g., reversible collapse) in response to small changes in environmental factors such as pH, temperature, or ionic strength [9]. Furthermore, the nucleoid was shown to undergo cycles of compaction-decompaction under the manipulation of crowding conditions [10], in line with the proposed switch-like conformational transition model for chromatin folding [11]. The regulatory role for “pre-programmed” phase transitions [9] was suggested by observations indicating that the nucleoid structure and gene expression are interdependent [6,12,13,14,15,16]. However, the tight coupling of chromosome structural transitions with gene expression dynamics appears at variance with the notion of gene expression as a fundamentally “noisy” stochastic process [17]. The deterministic average gene expression pattern observed at the cell population level emerges gradually from noisy single-cell expressions [18]. Yet, even though the gene expression patterns in individual bacterial cells might appear stochastic, the chromosomal gene order is not. Furthermore, temporally the gene transcription is correlated with the gene order along the OriC-Ter axis at the population level [12,19] and with the timing of gene replication at a single-cell level (see below).

## 3. Gradients of Regulators

At the population level, the bacterial cells can display clearly deterministic behavior such as chemotaxis [20] or quorum sensing, implying the ability of bacterial cells to monitor their density and adjust their collective behavior. In quorum sensing, a growing cell population produces increasing amounts of an autoinducer (AI), which ultimately attains concentrations that eventually turn on multicellular behavior. In principle, this phenomenon is akin to intracellular accumulation of the stringent response regulator guanosine (penta)tetraphosphate (p)ppGpp [21], producing a concentration spike on a shortage of resources and coordinating the transition to the stationary phase by interacting with various metabolic systems. Most importantly, ppGpp modulates the sigma-factor composition and thus, the promoter recognition specificity of the RNA polymerase (RNAP) holoenzyme [22,23,24,25]. In *E. coli,* ppGpp not only regulates the growth phase transitions but also the growth rate [26]. Although both the quorum-sensing autoinducers and ppGpp are continuous variables (as they are produced in various concentrations), their coordinating effects on cellular behavior likely depend on attaining particular threshold levels. Indeed, the *E. coli* cells demonstrate discretely calibrated responses to a gradient of ppGpp concentration [27]. The existence of such discrete, temporally distinguishable metabolic states is in line with the proposed discontinuous transitions of the *E. coli* phenotype during the growth cycle [28] and with the observed inter- and intra-cellular diversity of populations [29].

**Figure 1:**
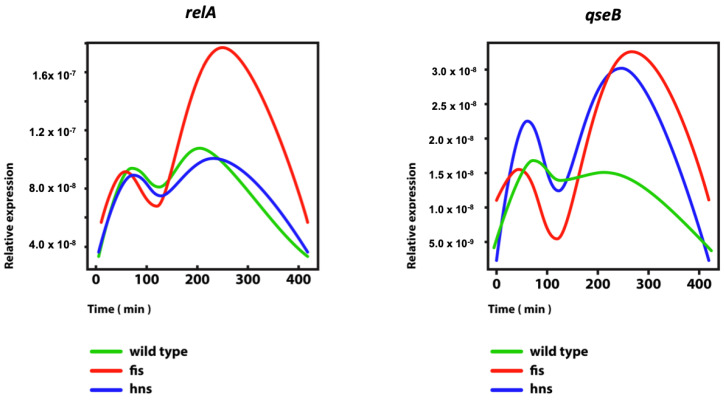
Regulation of the gene expression of ppGpp synthetase *relA* and the quorum-sensing regulator *qseB* in growing wild-type *E. coli* cells and mutant derivatives lacking the chromatin-shaping proteins FIS and H-NS. Abscissa—time (min) after inoculation of stationary cells in fresh medium. The time intervals 0–100’, 100–300’, and >300’ contain approximately the early (lag), middle (exponential), and stationary phases, respectively [12]. Ordinate—relative expression in arbitrary units. The color of the curves indicates the genetic background. The *Escherichia coli* CSH50 overnight cultures were inoculated at an initial OD600 of 0.1 in rich double-yeast-tryptone (dYT) medium and grown in a fermenter under constant pH 7.4 and high aeration (5 L air per min) at 37 °C for 7 h (420 min). Samples for RNA-seq were taken at intervals after inoculation as indicated and immediately dissolved in ice-cold ethanol–phenol (5% phenol) solution to prevent mRNA degradation. RNA was extracted using the RNeasy Mini kit (Qiagen, Hilden, Germany) and treated with Turbo DNase (Life Technologies, Carlsbad, CA, USA). Subsequent rRNA depletion was carried out using the MicrobExpress kit (Life Technologies), and 0.5 μg of enriched mRNA of each sample was subjected to RNA-seq (Illumina HiSeq 2000, Illumina, San Diego, CA, USA). In this figure and Figure 2 and Figure 3 time-resolved RNA-seq data of *E. coli* wild-type, *fis,* and *hns* mutant strains can be accessed via GSE65244 (NCBI Geo database).

Importantly, the genes involved both in quorum sensing and ppGpp synthesis, are regulated by DNA supercoiling and abundant nucleoid-associated proteins (NAPs) (Figure 1) [30,31,32]. The latter are acting both as determinants of chromatin architecture and global regulators of transcription [33]. The dynamic constraints of DNA supercoils by NAPs are, by and large, responsible for rendering the chromosome structure and gene expression interdependent. Both the abundance and composition of NAPs vary continuously forming temporal concentration gradients during the growth cycle [34,35,36], whereas their expression is in turn, regulated by ppGpp and/or by DNA supercoiling [1,37,38,39,40,41,42]. DNA supercoiling modulates not only the gene expression but also the efficiency of DNA binding by NAPs [43,44,45,46], whereas ppGpp can modulate the NAP-binding effect by protein modification [47]. More compellingly, the NAPs, DNA topoisomerases, and transcription machinery components are interconnected within an overarching homeostatic network. This network is involved in sensing the environmental changes and adjusting accordingly the chromosomal DNA superhelicity and gene expression during the growth cycle [1,8,38].

## 4. DNA Supercoiling Gradient and Genomic Sequence Organization

DNA supercoiling itself is a continuous variable directly responding to environmental changes [48,49,50,51]. What is the physical basis of this capacity? According to the classic physics definition, continuity finds its expression in the laws of nearby action, connecting only the values of physical quantities at space–time points in the immediate vicinity of one another. In the chromosomal DNA polymer, this “continuity” is afforded by the physical connectivity of the nucleotide sequence, where the “laws of nearby action” are embodied in stacking interactions between the DNA base pairs “in the immediate vicinity of one another”. Importantly, these local interactions can be modulated by supercoiling [52].

How is the spatial gene order translated into the temporal gene expression pattern? The coupling of temporal gene expression to spatial gene order is facilitated by the physical continuity of the chromosomal DNA polymer on the one hand and its topological closure on the other. Topological closure imparts the ability to directionally transfer the information over distance [53], which can be facilitated by DNA-bending NAPs [54,55,56,57]. The sequential character of gene expression implies directionality, which most readily can be associated with the process of replication, proceeding bi-directionally along both replichores from OriC toward Ter. Indeed, it was observed that gene transcription is temporally coupled to the event of gene replication during the bacterial cell cycle [58,59]. The link between gene replication and gene expression depends on the coupling of the transient supercoiling imbalance and supercoil diffusion induced by translocating replisomes [60,61] to gene transcription [62,63]. In addition to replication, the transcription of multiple exceptionally strong ribosomal RNA operons oriented from OriC toward Ter induces negative and positive supercoils, respectively, in the wake and ahead of the translocating RNAPs [64]. This directionally opposite diffusion of negative and positive supercoils potentially generates a global topological asymmetry along the chromosomal OriC-Ter axis. In *E. coli*, this imbalance is enhanced by a significantly (5–10 times) higher frequency of gyrase binding sites around the chromosomal OriC pole compared to Ter [6,14,65,66]. Furthermore, this diffusion of supercoils could facilitate the differential binding of regulatory proteins depending on their preference for binding, e.g., the strongly untwisted DNA emerging immediately behind the trailing end of the transcribing RNAP, the negatively writhed DNA formed further upstream, or the overtwisted/positively writhed DNA accumulating ahead of the translocating enzyme [67]. Importantly, the genome of *E. coli,* as well as other Gamma-proteobacteria, is characterized by a striking conserved sequence organization demonstrating an OriC-Ter gradient of DNA thermodynamic stability [12,14]. This finding together with the observed OriC-Ter gradient of gyrase binding sites led to the proposal that in addition to the temporal gradient of global superhelicity varying with available metabolic energy during the growth cycle [68], there exists a spatial gradient of superhelicity extending along the OriC-Ter axis of the chromosome [6]. Indeed, a chromosomal gradient of supercoiling has been detected in stationary *E. coli* cells [69] and recently, using more refined techniques, also in actively growing cells [70].

## 5. Role of Local Sequence Organization

In a study combining electron cryo-tomography with biochemical analyses of DNA minicircles, it was demonstrated that depending on the supercoiling level the DNA can adopt a range of distinct conformations [71]. However, the response to superhelical stress also depends on the DNA sequence organization [53]. In particular, local DNA sequence organization was shown to be determinative for transition dynamics between alternative 3D structures demonstrating coordinated, long-range interactions within a common topological domain [72,73,74,75]. These long-range interactions vary as a function of superhelical density and likely arise from the competition of discrete transitions for the free energy of negative supercoiling [72]. Thus, although the variation of superhelical density alone can determine the distinct 3D conformations of DNA [71], the available supercoil energy can also specify various DNA structures depending on the local sequence organization [53,76].

On a local scale, the DNA sequence organization of individual gene/operon promoter regions demonstrates different helical periodicities associated with particular responses to supercoiling, as well as enrichment for sequences stabilizing alternative DNA structures and intrinsically curved DNA [77,78,79,80]. Intrinsically curved DNA sequences were shown to facilitate the pinning of plectonemic supercoils upstream of the promoter [81]. Furthermore, the variations in canonical promoter elements such as the G/C content of the discriminator sequence (the sequence between the −10 hexamer and the transcription start site) and the length of the spacer between the −10 and −35 hexamers, were shown to be determinative for the promoter supercoiling response [40,42,82,83,84,85,86]. For example, the “stringent” promoters (such as stable RNA promoters) that are, respectively, down- and up-regulated by ppGpp and negative supercoiling are characterized by G/C-rich discriminators, short (16 bp) spacers, and suboptimal −35 hexamers as well as anisotropically bendable upstream activating sequences (UAS) forming coherently bent DNA microloops associated with RNAP [55,87,88]. Overall, the more A/T-rich and more G/C-rich sequences are associated with a response to low and high levels of negative superhelicity, respectively [42,84,85].

## 6. Topological Domains

In addition to the DNA sequence organization ordered by thermodynamic stability, the circular bacterial chromosome appears organized in hundreds of topologically isolated domains of ~10–20 kb in size [89,90,91], the boundaries of which were found to be modulated by mutations in DNA gyrase [90], metabolic genes, and genes encoding the NAPs, e.g., FIS and H-NS [92,93]. High-resolution Hi-C mapping of the *Caulobacter crescentus* chromosome suggested the existence of multiple, largely independent domains comprising supercoiled plectonemes arrayed into a bottlebrush-like fiber [94], consistent with the formation of higher-order plectonemes (hyperplectonemes) observed in vitro [44]. Thus, it appears that while the local sequence organization is determinative for the promoter supercoiling response, on a genome-wide scale the thermodynamically variable DNA sequence is organized into topologically isolated domains of apparently regular size. Such a confinement of genes/operons within topologically isolated domains suggests a mechanism for the independent yet coordinated regulation of promoters by modulating the available torque. This notion is consistent with the variable genomic patterns of the transcriptional supercoiling response observed during the bacterial growth cycle [31]. Topological domains could also mediate the communication between different RNAP molecules by allowing the transmission of information along the DNA as an available torque for promoter opening [95].

## 7. Modulation of the Transcriptional Supercoiling Response by NAPs

It was observed that the changes in supercoiling induced in growing bacterial cells under the influence of drugs modulating the topoisomerase activities produce distinct, long-range transcription patterns [40,65,96,97]. The supercoiling responses of genomic transcription were found to vary with the growth phase and be distinctly modulated by NAPs, such as FIS, H-NS, IHF, and HU [31,57,66,96]. Although both FIS and H-NS constrain negative supercoils [43,98], it was shown that the binding of FIS at helically phased sites in the UAS of stable RNA promoters stabilizes coherently bent DNA microloops buffering the promoter activity on deviations from optimal superhelicity [83,88,99]. Another highly abundant NAP, HU, appears involved both in the maintenance of the global supercoiling level [45,46,100] and in the topological buffering (insulation) of transcription units [101]. The cells lacking HU demonstrate a loss of higher-order structural features of the nucleoid such as transcription foci as well as produce an aberrant genomic transcription pattern [66]. In the plant pathogen ‘*Dickeya dadantii*’, deletion of the abundant NAP IHF leads to the spatial expansion of the transcriptional supercoiling response from the OriC and Ter poles along both chromosomal arms [57]. In both *E. coli* and *D. dadantii*, such spatial patterns of the supercoiling response, induced by environmental stress or topoisomerase poisons/inhibitors and modulated by NAPs, have been variably described as gene proximity networks [102], extended functional domains [12], stress-response domains [96], or coherent domains of transcription, also known as CODOs [103].

## 8. Coupling of DNA Structure to Function Using Two Types of DNA Information

The CODOs (formerly “functional domains”) [12] emerge as growth condition-dependent, transient, spatially extended gene expression patterns. These spatially confined patterns are thought to manifest the structural-functional organization of the bacterial genome. In *D. dadantii,* the CODOs comprising the thermodynamically variable DNA sequences are associated with distinct genetic traits; in other words, the CODOs, which emerge in various constellations depending on the applied stress, integrate the chromosomal transcriptional response to the stress-induced peculiar changes of supercoiling with the expression of stress-specific virulence and adaptation genes [96,103,104]. This coupling of the DNA physicochemical properties and the supercoiling response with particular genetic function within the CODOs, underscores once more the role of genomic sequence organization (i.e., the spatially ordered distribution of DNA thermodynamic stability) in coordinating the global transcriptional response. Also, in *Streptococcus pneumoniae* the sequence composition of chromosomal domains harboring the distinct adaptation and virulence traits was found to be determinative for peculiar supercoiling response [105,106], again underscoring the role of genomic sequence organization in coordinating the bacterial genetic response. In *E. coli*, this strategic coupling of the supercoiling response and the genetic function in genomic sequence organization is made conspicuous in the selective enrichment of the relatively G/C-rich OriC and relatively A/T-rich Ter chromosomal poles for anabolic and catabolic genes, respectively [3,107]. More compellingly, the bacterial genomic sequence organization reveals how during the growth cycle the environmentally determined availability of superhelical energy can be fittingly coupled to a genetic adaptive response. In *E. coli* this directional supercoiling response is encoded in and enabled by the peculiar genomic sequence organization along the OriC-Ter axis; in other examined bacteria (especially in pathogens having a relatively complex lifestyle) the genomic organization patterns may be more nuanced yet represent variations on a common theme [103,104]. As mentioned above, in general, both the local and global transcriptional responses to supercoiling vary, with relatively G/C-rich and G/C-poor sequences responding to high and low levels of negative superhelicity, respectively [42,84].

The patterns of the sequence-dependent supercoiling responses of genomic transcription demonstrate that in addition to static genetic information (digital code), the chromosomal DNA polymer also provides dynamic information manifested in the variable distribution of the available DNA torque. The latter determines the organization of functionally meaningful spatial transcript patterns in the genome. Essentially, this dynamic information depends on the distinct thermodynamic stabilities of the consecutive DNA base steps, which overlap and thus provide information of a continuous or analogue type [52,107]. The DNA analogue information encoded in various arrangements of base steps stabilizing distinct DNA conformations facilitates the binding of proteins involved in DNA transactions including transcriptional control [108,109,110,111]. More specifically, the genome encodes both the amino acid sequences of DNA-binding proteins and enzymes governing DNA transactions, as well as the dynamic, supercoiling-dependent structures, serving as recognition signatures recruiting these enzymes and DNA-binding proteins to particular genomic loci [3]. This notion is consistent with the proposed evolutionary “multiplexing” of DNA genetic and structural information into the same molecular context [112]. Thus, by operating with these two—discontinuous (digital) and continuous (analogue)—types of information interwoven in the very same genomic sequence, the DNA appears to communicate with itself. We propose that this “self-communication” represents the global feedback mechanism endowing the growing cell population with the capacity to monitor its status quo, a capacity that is reflected, in part, in the interdependence of chromosome structural dynamics and spatial patterns of gene expression [6,15,16].

## 9. Role for Changing Chromosome Configuration in Organizing Genomic Transcription

The bacterial growth cycle appears as an intrinsically ordered process following a “programmed” as it were, passage of the population through successive, physiologically distinct states or “growth phases” associated with transitions in nucleoid structure [35,113,114,115]. Notwithstanding the existence of evolutionarily pre-programmed phase transitions in nucleoid structure, this sequential order during the bacterial growth cycle could also be produced by the continuous adjustment of the physiological state to changing growth environments and thus, be largely determined, or better to say, triggered by the latter. What we touch upon here is essentially a “nature versus nurture” issue. In this respect, a relevant observation is that environmental factors can be crucial in determining the genetic capacity of *E. coli* to vary traits qualitatively [116]. However, the question we ask here is how the environmental change is translated into physiological alteration and, first and foremost, into an adequate transcriptional response.

Spatiotemporal organization of the transcription “program” governing the growth in *E. coli* has been described previously in considerable detail [6,12,117]. Briefly, during the *E. coli* growth cycle, the transcriptional activation of the chromosomal OriC and Ter poles occurs consecutively [12]. A similar, yet more nuanced pattern was observed in *Salmonella enterica* [118]. On nutritional shift-up, under conditions of high negative superhelicity, the transcription of the relatively G/C-rich chromosomal OriC pole is activated, whereas that of the Ter pole is repressed [12]. Conversely, on the transition to the stationary phase associated with the global relaxation of the DNA, the relatively A/T-rich Ter pole is activated, whereas the OriC pole is repressed. Since it is assumed, as mentioned above, that the chromosome configuration and genomic transcription are interdependent [6,15,16,119,120], the successive activation of OriC and Ter poles likely reflects the coordinately changing configuration and genetic activity of the chromosome, which is associated with the global redistribution of RNAP in the nucleoid [118,121]. In this regard, the non-random distribution of the binding sites of the NAPs and especially of the DNA gyrase and major RNAP sigma factors along the OriC-Ter axis that were observed in the *E. coli* genome [6,14], are suggestive. This gross transition of chromosome configuration during the growth cycle was proposed to act as a topological device governing the “growth program” by converting the available metabolic energy into the growth-phase-dependent gene expression pattern, which seems connected via the metabolites to replication [2,6,122]. Ultimately, it appears that the spatial organization of DNA thermodynamic stability in bacterial genomes serves the purpose of the temporal coordination of chromosome structural dynamics and genetic response [107].

## 10. Coupling of DNA Topology, NAP Binding Effects, and Holoenzyme Sigma-Factor Composition: Major Regulatory Events during the *E. coli* Growth Cycle

On nutritional shift-up, the expression of the *gyrA* and *gyrB* genes encoding DNA gyrase—an enzyme introducing negative supercoils into the DNA in an ATP-dependent manner,—increases [123,124] concomitantly with an increase in the ATP/ADP ratio [125,126,127]. It is likely that at the chromosomal OriC pole negative superhelicity rapidly attains high densities of σ~−0.07 to −0.08, consistent with both the enrichment of the OriC pole for the gyrase binding sites and the preferential binding of gyrase downstream of the strongly transcribed genes organized predominantly around OriC including the exceptionally strong rRNA operons [66,128]. Increased negative superhelicity both facilitates replication initiation and strongly activates the OriC-proximal *fis* gene expression [37,39,129], whereas the accumulation of FIS in turn maintains activated ribosomal RNA transcription [130]. Structurally, the increase in negative superhelicity leads to a branching of plectonemically coiled DNA, thus multiplying the tightly bent apical loops and facilitating the wrapping of DNA by the RNAP σ^70^ holoenzyme [88,131,132,133], which prefers highly supercoiled templates for transcription [134]. Notably, the RNAP σ^70^ holoenzyme demonstrates an OriC-Ter gradient of binding site frequency distribution correlating with that of the gyrase binding sites [6]. Concomitantly with activation of the *fis* gene, the expression of the *rpoZ* gene encoding the RpoZ (ω)subunit of RNA polymerase is also strongly increased [3,124]. RpoZ stabilizes the polymerase σ^70^ holoenzyme assembly [135] and is also directly involved in mediating the response to ppGpp [23,136]. FIS activates the *hupA* gene [137] such that at the early growth stage, the high levels of negative superhelicity and the RNAP σ^70^ holoenzyme coexist with increased levels of the two major “early” NAPs—FIS and HUα. High FIS levels boost rRNA synthesis, whereas HU supports the organization of rRNA operons in “transcription factories” engaging hundreds of RNAP molecules [66,138]. The latter effect requires high levels of negative superhelicity [121]. Accordingly, both FIS and HU stabilize negative supercoils [43,139,140].

On transition to the stationary phase associated with a shortage of resources, the rapid increase in ppGpp levels (ppGpp spike) decreases both rRNA transcription and the supercoil density around OriC, thus precluding replication initiation [141]. The *gyr*A/*B* gene expression and the gyrase levels subside [123] and the total superhelicity drops to σ~−0.03. The down-regulation of the chromosomal OriC pole at this stage is likely supported by the binding of the global repressor H-NS and the “late” NAPs such as Lrp and Dps [6,142], whereas the activation of the Ter pole is attributable to the accumulation of the RNAPσ^S^ holoenzyme and IHF, the binding sites for both of which are strongly enriched around the chromosomal Ter pole [6]. Notably, the “late” NAPs as well as the RNAPσ^S^ holoenzyme, preferentially bind relaxed DNA [3]. Thus, in the stationary phase, the RNAPσ^S^ holoenzyme responsible for the maintenance function predominates, whereas the globally relaxed DNA is organized by the binding effects of three abundant NAPs including IHF, which stabilizes planar bends without the substantial constraints of negative superhelicity, Lrp, which constrains positive supercoils, and Dps, which packages the relaxed DNA in a protective crystalline lattice [115,143].

What happens to the nucleoids of cells enriched for these abundant stationary-phase NAPs on nutritional shift-up? After nutritional shift-up, the compaction state of the nucleoid undergoes marked alterations adopting a more open conformation [144], presumably due to competition between the early and late growth stage NAPs, respectively, FIS and Dps [142,145]. The DNA binding of IHF and Dps was shown to depend on environmental factors [146]. Under conditions of the increase in DNA negative superhelicity after nutritional shift-up, both IHF and Dps likely start to dissociate from genomic binding sites, perhaps accelerated in part by “facilitated dissociation”—a general mechanism thought to modulate gene expression by assisting in the local removal of DNA binding proteins from cognate sites [147,148,149]. However, IHF remains stably bound at the chromosomal origin of replication, its dissociation being prevented by the replication initiator protein DnaA cooperating with IHF in initiating chromosomal replication [150]. This latter process is facilitated by FIS protein produced at high levels on nutritional shift-up [151].

## 11. Genomic Transcription during the Bacterial Growth Cycle Is Steered by Supercoil Energy

Changes in DNA supercoiling are assumed to mediate the transmission of environmental changes to the chromosome, responding not only to the altered availability of metabolic resources during the growth cycle [68] but also to the various stress factors including suboptimal oxygen tension, temperature, and osmolarity [125,152,153,154]. This mediation by supercoiling is due to the capacity of the DNA double helix to sense the environmental conditions and to respond by adjusting accordingly the superhelical density on the one hand, and by channeling the available superhelical energy into corresponding gene expression patterns on the other. We have argued that during the bacterial growth cycle, changes in DNA topology modulate the binding of DNA architectural proteins and the activity of transcription machinery concertedly, resulting in orchestrated directional alterations of chromatin architecture and gene expression. Several lines of evidence are consistent with this notion.

First, the continuous alteration of DNA superhelical density during the growth cycle is dependent on the availability of nutritional resources [68]. As in the progression of the population along the growth cycle, the superhelical density decreases, the tightness of apical loops and DNA interwindings also decreases, and so the accessibility to the RNA polymerase changes. As mentioned above, from the early exponential phase to the early stationary phase dominated by the RNAPσ^70^ holoenzyme, the number of total superhelical turns per 1 kb would reduce from ~8 to ~3, with a reduction in unconstrained turns likely to be greater and hence, the available DNA superhelical torque lower. Under these conditions, the RNAPσ^S^ holoenzyme preferring relaxed DNA substrates for transcription becomes active [25,134]. At the early growth stage the RpoZ-dependent stabilization of the RNAPσ^70^ holoenzyme facilitates the utilization of negatively supercoiled templates, whereas the lack of RpoZ leads to a global DNA relaxation and an increased activity of the RNAPσ^S^ holoenzyme, switching the global transcription preferences to the utilization of the relaxed DNA templates [155]. Importantly, the impact of accumulated σ^S^ could not be detected until the superhelical density of the reporter plasmids subsided to relatively low levels (∆Lk of ~3–4 between the late exponential phase and the early stationary phase, where the σ^S^ impact was observed). It was thus inferred that the variations in DNA supercoiling as a function of the growth phase act as a checkpoint, precluding the shift from the RNAPσ^70^ to the RNAPσ^S^ transcriptional machinery until the growth conditions become unfavorable enough to cause entry into the stationary phase [156].

Second, the superhelical density and the supercoiling response of the genomic transcription change coordinately both during the growth cycle of *E. coli* [31] and during the circadian cycle of gene expression in cyanobacteria. A combination of topological analyses with transcriptomics data in *Synechococcus elongatus* suggested that each topological state corresponded to a unique state of gene expression, indicating that supercoiling plays a primary role in regulating circadian gene expression [157]. Notably, the DNA sequence characteristics of genes monotonically activated and repressed by chromosomal relaxation during the circadian cycle were similar to those of the supercoiling-responsive genes in *E. coli*.

Third, the sequential activation of sets of primary and downstream regulatory genes was observed in response to the long-term supercoiling imbalance achieved by modulating the *topA* gene expression in *Streptomyces coelicolor*, whereby increased negative superhelicity modified the levels of topoisomerases and NAPs coordinately [40]. Another relevant observation comes from experimental evolution studies, which identified mutations in genes encoding the NAPs and topoisomerases. These mutations induced inheritable adaptive changes of supercoiling and also provided fitness gains, thus revealing the pivotal role of NAPs and topoisomerases in organizing the global transcription program during adaptation [158,159].

Fourth, supercoiling can impose directionality by rendering the structural transitions in DNA both deterministic [160] and coordinated [72,73,74,75], whereas the nucleoprotein structures stabilized by NAPs have been implicated in the directional channeling of torsional energy toward the transcription initiation sites [55,161,162]. Available data indicate that the NAP-dependent alterations of gene expression during the growth cycle involve directional and coordinated transitions in the composition of regulatory nucleoprotein structures associated with gene promoter regions [163,164,165,166]. These directional transitions of nucleoprotein complexes associated with individual gene promoters are paralleled by the coordinated redistribution of transcription machinery during the growth cycle at the global scale of the entire chromosome [118,121].

**Figure 2:**
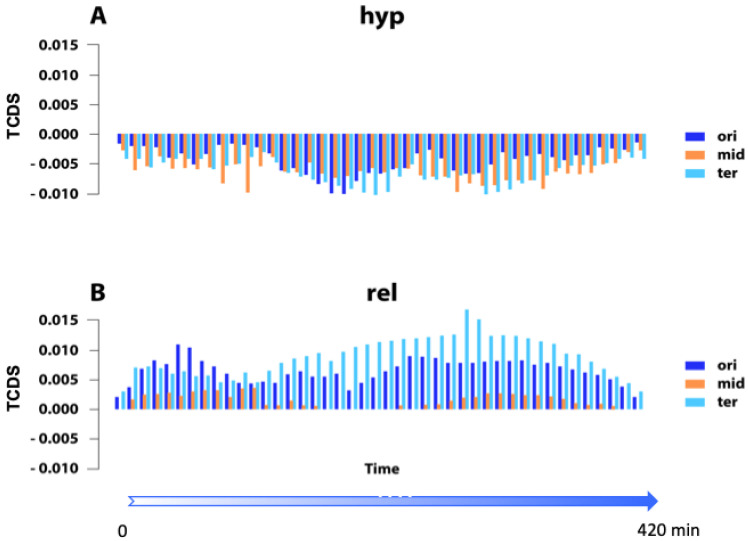
TCDS strength measured at the gene promoter regions during the growth cycle of *E. coli* grown in batch culture. The entire dataset was divided into three equally sized sets of OriC-proximal (ori), intermediate (mid), and Ter-proximal (ter) genes. TCDS was determined assuming a 10 kb range as previously described [170], whereby the impact of TCDS is exponentially decreasing with distance to its originating gene. (**A**) Average TCDS of neighboring transcription for *hyp* genes [31] activated under conditions of hyper-negative DNA supercoiling. (**B**) Average TCDS of neighboring transcription for *rel* genes [31] activated under conditions of DNA relaxation. TCDS was measured at 10 min intervals during the entire growth cycle (0–420 min) using the RNA-Seq data from [12]. Abscissa—time (in minutes) after inoculation of cells in the fresh medium. Ordinate—strength of TCDS; positive values indicate impact of positive superhelicity, negative values indicate impact of negative superhelicity. For growth conditions see legend in Figure 1.

Finally, it is noteworthy that the regulation of gene transcription by supercoil energy is also modulated by gene organization in the genome. The processes of DNA supercoiling and transcription are interdependent [167] and in addition to the specific regulation of gene promoters, global transcriptional responses to changes in DNA supercoiling depend on constraints imposed by the local orientation of genes and the supercoil diffusion induced by the transcription process itself [168,169,170,171]. As mentioned above, transcription generates negative and positive supercoils, respectively, upstream and downstream of the translocating RNAP and this transcription-coupled diffusion of supercoils (TCDS) modulates the activity of neighboring gene promoters. The TCDS effect exerted on a particular gene depends on the mutual orientation (either convergent, divergent, or tandem) of its surrounding transcription units. For example, a gene embedded between two divergently oriented transcription units will experience high levels of negative superhelicity (negative TCDS), and vice versa in the case of flanking convergent units, high positive superhelicity (positive TCDS). Although the transcriptional regulation shaped by local genomic architecture can be modulated by changes in global supercoiling and by NAPs [57], these local constraints provide additional means for fine-tuning the supercoiling-dependent impacts on gene expression during the growth cycle. In particular, the measurement of TCDS during the growth cycle of *E. coli* shows that the genes responding to high negative superhelicity (*hyp* genes) also experience negative TCDS from their neighbors, whereas the genes responding to DNA relaxation (*rel* genes) experience positive TCDS (Figure 2). TCDS varies noticeably with growth time and chromosomal region. These data are fully consistent with the notion of local genomic architecture providing additional means for modulating and fine-tuning the effects of changing DNA superhelicity on gene expression during the bacterial growth cycle.

## 12. Spatially Shifting Superhelicity Optimum Determines the Temporal Gene Expression

There is little doubt that supercoiling regulates global gene expression, but this by itself does not explain how the spatiotemporal order of gene expression is established. The latter has been correlated with gene replication [59] and thus with the cell cycle, but what about the gene expression order during the growth cycle of the bacterial population? Although Figure 2 shows a variable pattern of TCDS during the growth cycle, this pattern reflects the changing impact of ongoing transcription on neighboring supercoiling-sensitive genes in distinct chromosomal regions but does not reveal much about the spatiotemporal order of gene expression.

It is conceivable that the genomic sequence organization, in conjunction with the temporal gradient of supercoiling, determines the spatiotemporal gene expression order during the bacterial growth cycle. As mentioned above, the genomic sequence of *E. coli*, and Gamma-proteobacteria in general, demonstrates a gradient of DNA thermodynamic stability (approximated by G/C-richness) along the OriC-Ter axis of the chromosome, whereas in general, the relatively more A/T-rich and more G/C-rich sequences respond to low and high levels of superhelicity, respectively [42,84,85].

**Figure 3:**
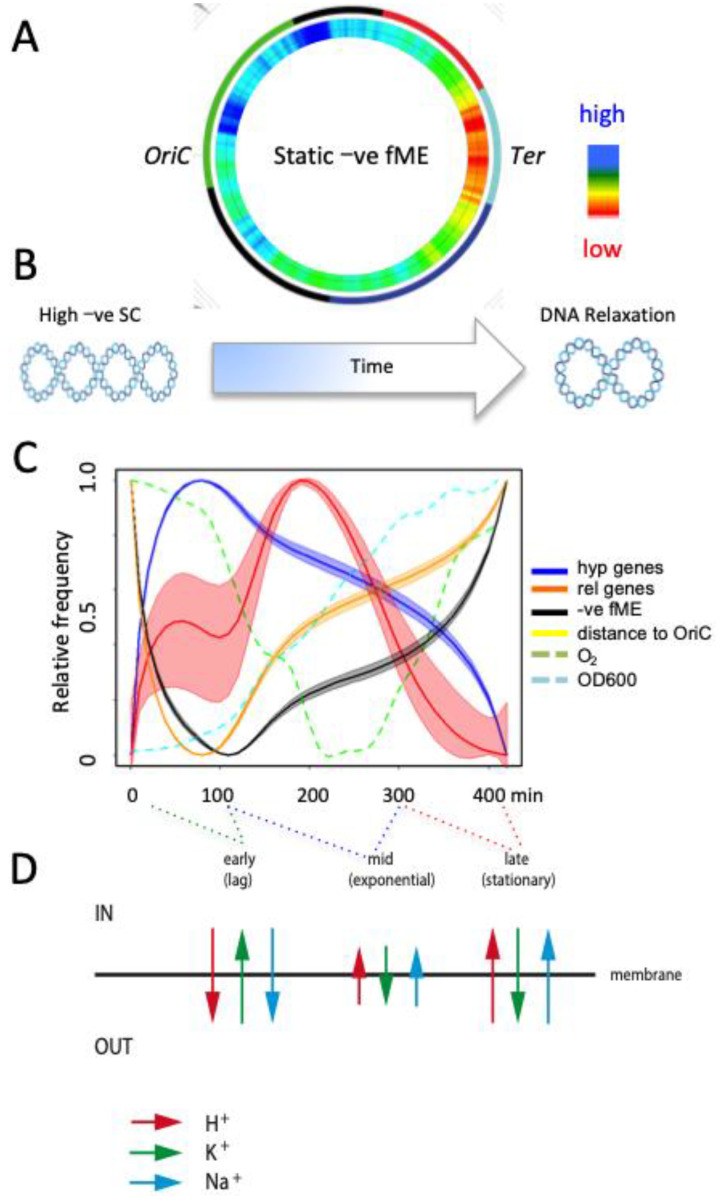
The proposed model of the coupling of the temporal gradient of superhelicity with spatial gradient of DNA thermodynamic stability as a device determining the spatiotemporal gene expression. (**A**). The *E. coli* genomic wheel with macrodomains depicted on the outer ring: green—Ori; dark blue—left; red—right; black—the left and right non-structured domains; light blue –Ter macrodomain. On the genomic wheel, the distribution of free negative melting energy (-ve fME, inner ring; 500 kb scanning window) is color-coded in blue (for high) and red (for low). The -ve fME was defined using the parameters of Santa Lucia [173]. The positions of OriC and Ter are indicated. (**B**). The horizontal arrow indicates the passage of time after the nutritional shift-up and associated decrease in global DNA negative superhelicity (-ve SC) during the growth cycle. (**C**). The plot showing the temporal variation in different parameters in the gene expression profile obtained during the growth cycle from inoculation of cells (at 0 min) to the late stationary phase [12]. The different curves were normalized (0;1) to compare them in one plot. Expression values of genes responding to high negative supercoiling (*hyp* genes) and DNA relaxation (*rel* genes) are normalized to the expression of all genes. Negative melting energy and distance to origin were averaged over all genes weighted by their expression. Because melting energy is by convention expressed as a negative ∆G value, high melting energies have a lower (more negative) numerical value, i.e., the lower the negative melting energy value the higher the G/C content. Note that the high negative melting energy values correlate with small distances to replication origin. The envelopes of the curves indicate the standard deviation at 10% random remapping of the expression patterns to genes. The optical density and partial oxygen pressure, respectively, are indicated by the dashed blue and green lines. For growth conditions see legend in Figure 1. Abscissa—time in minutes after inoculation. Ordinate—relative frequency in arbitrary units. (**D**). Temporal changes of ion composition and intracellular pH. IN and OUT indicate the intra- and extracellular compartments. Arrows indicate directional (influx/efflux) changes. The colored dashed lines drawn between the panels (**C**) and (**D**) correlate the growth stages indicated in (**D**) to time intervals in (**C**). For details see the text.

In particular, the G/C composition of the promoter discriminator sequence was shown to be determinative for the supercoiling response [83,85]. Individual gene promoters for which the supercoiling response optima were studied in detail, are shown in Table 1. Although the sample of supercoiling-sensitive promoters provided in Table 1 is far from comprehensive, it shows a trend suggesting that the closer the promoter is located to OriC, the higher the optimum superhelical density for its transcription, consistent with the gradual decline in the average DNA thermodynamic stability (-ve fME, ~G/C content) as a function of distance from OriC (see the genome wheel in Figure 3A). This general trend—a positive correlation between the transcriptional response to high negative superhelicity, high negative melting energy content (~G/C-richness), and proximity to OriC of the genes—is corroborated by time-resolved analyses of global genomic transcription during the growth cycle (Figure 3C).

We have argued that the process of replication as well as of ribosomal gene transcription proceeding directionally from OriC toward Ter imposes a supercoiling asymmetry with higher negative superhelicity accumulated at the OriC pole of the chromosome [6,66]. At the early growth stage under conditions of high negative superhelical density, the OriC proximal G/C-rich sequences are likely to be transcribed optimally. However, it is conceivable that the gradual decrease in the global superhelical density during the growth cycle [68] would shift the optimum of the promoter supercoiling response toward genomic regions with lower average G/C content and hence, away from OriC toward Ter. Thus, with the passage of time and the associated decrease in global negative superhelicity, progressively more OriC-distal genes with lower G/C content and lower optima of superhelical density for transcription would become maximally active. We propose that the coupling of the temporal growth-phase-dependent gradient of chromosomal DNA supercoiling with the spatial gradient of genomic DNA thermodynamic stability along the chromosomal OriC-Ter axis acts as a timing chain determining the spatiotemporal order of gene transcription during the bacterial growth cycle (Figure 3).

## 13. The Temporal Gradient of Superhelicity Reflects the Gradient of Ion Composition and Intracellular pH

The temporal gradient of superhelicity apparent during the growth cycle and its correlation with the energy requirement for promoter opening, suggest that both these effects are a response to changes in energy availability. A primary response to shift-up is an almost immediate doubling of the intracellular energy charge from the stationary phase level [174]. On a similar time scale, there is a rapid influx of K^+^ mediated by the proton-dependent K^+^ transporting P-type ATPase accompanied by an efflux of H^+^ and Na^+^ resulting in an excess of K^+^ [175]. The net effect of these changes in the ion composition is an increase in intracellular pH [176]. The change in energy charge could also promote DNA gyrase activity [126]. During the subsequent growth cycle, the relative concentrations of K^+^ and Na^+^ are rebalanced [175] whereas the energy charge falls from the onset of the stationary phase [174], and the intracellular H^+^ concentration increases [177]. The observed changes in the intracellular ion composition during the growth cycle are likely relevant to transcriptional regulation and DNA compaction. The efficient in vitro transcription of supercoiled DNA is K^+^ (and not Na^+^) dependent [178], whereas during the stationary phase the relative DNA binding of the major NAPs, IHF and Dps, depends on the pH, K^+^, and Mg^++^ concentrations, with that of Dps being favored by low pH and low [K^+^] [146]. We suggest that the temporal gradient of superhelicity reflects a corresponding temporal gradient in the ion composition and intracellular pH, which in turn would influence energy availability via the F_0_F_1_ ATPase. Notably, on the *E. coli* chromosome the *atp* operon maps immediately adjacent to OriC.

## 14. Conclusions

Previously, we have argued that the central task of “translating” environmental signals into the appropriate physiological responses is carried out by the bacterial chromosome acting as a thermodynamic machine [2]. This is due to the capacity of the heterogeneous DNA double helix to sense environmental changes and adjust the superhelical density on the one hand and transform the superhelical energy into distinct genomic structures with associated gene expression patterns on the other. This transformation of supercoil energy into genetic information is enabled by the specific coupling of the DNA thermodynamic stability (essentially, DNA structural dynamics) with genetic function [12,104,107], revealing crosstalk between two different types of information interwoven in the genomic DNA sequence. Here, we propose that this double informational content of DNA, reflected partly in the interdependence of genomic expression and structural dynamics of the chromosome [3,15,120,179], enables perpetual monitoring of the physiological state, manifested in the changing genomic binding patterns of NAPs and RNAP sigma factors during the bacterial growth cycle [6,34,35,118,180]. By coupling the temporally changing ion composition, intracellular pH, and energy levels via DNA topology to the spatially shifting gene expression pattern in the genome (note that during the growth cycle the gene expression changes as a function of distance from OriC; Figure 3C), the bacterial population grown in batch culture could also measure the ‘traveled distance’ (i.e., its age) along the growth cycle. We propose that continuous feedback, coupling the genetic activity with the genomic distribution of superhelical energy during successive growth phases, has the potential to enable both self-monitoring as well as directional shifts adjusting the physiology to environmental changes. We suggest that genomic sequence organization is central to the realization of both these functions.

## Figures and Tables

**Table 1:** Optimal superhelical density for promoter activity as a function of distance from OriC (OriC at 3.92 Mbp; Ter at 1.59 Mbp).

Gene Promoter	Distance from OriC (bp)	Condition	Optimal * Superhelical Density (σ)	#References
*hisR*	62,500	In vitro	~−0.08 to −0.1	[172]
*rrnAP1*	120,000	In vitro	~−0.076	[99]
*fis*	508,500	In vitro & in vivo	~−0.07-to −0.08	[39]
*tyrT*	1,967,600	In vitro	~−0.05–0.06	[83]
*osmE*	2,097,900	In vitro	~−0.03–0.04	[156]

* The promoter activity declines on both sides of the indicated σ values.

## Data Availability

The time-resolved RNA-seq data of *E. coli* wild-type, *fis,* and *hns* mutant strains can be accessed via GSE65244 (NCBI Geo database).
